# *Bifidobacterium adolescentis* CCFM1447 effectively alleviates osteoporosis by enriching intestinal flora capable of vitamin D conversion

**DOI:** 10.3389/fnut.2025.1647671

**Published:** 2025-09-23

**Authors:** Xihua Yu, Gao Tian, Yi Wang, Liuruolan Li, Liming Huang, Yurong Zhao, Ling Feng, Yuhao Zhao, Haiqin Fang, Wenwei Lu, Shourong Lu, Hongchao Wang

**Affiliations:** ^1^Sinopharm Xingsha Pharmaceutical (Xiamen) Co., Ltd., Xiamen, China; ^2^Xiamen Key Laboratory of Maternal and Infant Health and Nutrition Products, Xiamen, China; ^3^State Key Laboratory of Food Science and Resources, Jiangnan University, Wuxi, China; ^4^School of Food Science and Technology, Jiangnan University, Wuxi, China; ^5^NHC Key Laboratory of Food Safety Assessment, Chinese Academy of Medical Science Research Unit (2019RU014), China National Center for Food Safety Risk Assessment, Beijing, China; ^6^National Engineering Research Center for Functional Food, Jiangnan University, Beijing, China; ^7^The Affiliated Wuxi People’s Hospital of Nanjing Medical University, Wuxi People’s Hospital, Wuxi Medical Center, Nanjing Medical University, Wuxi, China

**Keywords:** osteoporosis, intestinal flora, vitamin D, active vitamins, probiotics

## Abstract

**Introduction:**

Elderly individuals exhibit heightened susceptibility to osteoporosis, largely attributable to age-related declines in skin, liver, and kidney function. While vitamin D (VD) supplementation is common, its efficacy is often limited, necessitating reliance on pharmaceutical interventions. Research indicates that the intestinal flora significantly influences intestinal VD metabolism, with probiotic supplementation demonstrably impacting circulating VD levels.

**Methods:**

We employed an fecal fermentation model to screen bacterial strains. After introducing these strains into osteoporotic mice, we tested the mice’s serum and skeletal indicators. We then conducted a correlation analysis between the mice’s key intestinal microbiota and serum and skeletal indicators.

**Results and discussion:**

We identify *Bifidobacterium adolescentis* CCFM1447 for its capacity to elevate VD metabolite levels within fermented supernatants. It significantly elevated serum concentrations of 1,25-dihydroxyvitamin D. Furthermore, this intervention improved bone microarchitecture, evidenced by increased trabecular number and bone volume fraction. In addition, the intestinal flora of the osteoporotic mice was disturbed. CCFM1447 intervention increased the relative abundance of beneficial bacteria such as *Adlercreutzia equolifaciens*, *Akkermansia muciniphila* and *Pediococcus acidilactici*. And it is enriched with a part of strains that have the ability to transform VD such as Enterococcus faecalis and Pediococcus acidilactici. The above results suggest that *B. adolescentis* CCFM1447 may alleviate retinoic acid-induced osteoporosis symptoms by modulating the intestinal flora and increasing the level of active vitamin D.

## 1 Introduction

Osteoporosis is a disorder characterized by decreased bone mass and deterioration of bone microarchitecture. It collectively contributes to a heightened risk of fractures in affected individuals (1). Common clinical manifestations include skeletal pain, loss of stature, kyphosis, fractures, and impaired pulmonary function. Although calcium supplementation is considered a fundamental therapeutic strategy, it is insufficient as a standalone treatment, necessitating pharmacologic intervention tailored to the patient’s clinical profile.

Currently, the therapeutic arsenal for osteoporosis includes bisphosphonates, selective estrogen receptor modulators (SERMs), estrogen, and calcitonin. These agents are effective in promoting osteoblast activity while suppressing osteoclast-mediated bone resorption. Nevertheless, bisphosphonates are associated with renal toxicity and may induce or worsen hypocalcemia (2). Raloxifene, a SERM, has been linked to an increased risk of stroke. Estrogen therapy elevates the risk of endometrial malignancy in women with an intact uterus, and is associated with higher rates of gallstone formation and thromboembolic events (3, 4). Similarly, the RANKL inhibitor denosumab shares adverse effect profiles with bisphosphonates. Calcitonin use can provoke allergic reactions and, in rare cases, anaphylaxis; high doses may induce hypocalcemia and have been implicated in a potential increase in cancer risk (5). Importantly, none of these anti-fracture treatments are curative, and bone degeneration tends to recur following cessation of therapy. Alarmingly, 85% of patients discharged after hip fracture repair do not receive continued anti-fracture management or pharmacologic therapy (6–8). This underscores the pressing need for new treatment modalities that are both effective and free from long-term complications.

Vitamin D (VD) is a vital micronutrient necessary for normal bone physiology, growth, and mineral ion regulation. Its significance in maintaining skeletal integrity and mineral balance is well-documented. Epidemiological studies have also linked VD deficiency to a heightened risk of various diseases, including musculoskeletal, metabolic, cardiovascular, autoimmune, malignant, and infectious conditions (9–11). The biologically active form of VD, 1,25-dihydroxyvitamin D [1,25(OH)₂D], functions as a ligand for the vitamin D receptor (VDR), playing a critical role in calcium homeostasis and immune modulation (12). Supplementation with VD is particularly beneficial for children and older adults, contributing to improved muscle strength, enhanced postural balance, and overall bone health.

Individuals with osteoporosis often exhibit impaired VD metabolism. As a fat-soluble vitamin, VD can be synthesized endogenously through dermal exposure to ultraviolet radiation and acquired exogenously through diet (13). To become biologically active, VD undergoes two hydroxylation steps: converting it to 25-hydroxyvitamin D [25(OH)D] in the liver (14), and then in the kidneys to form 1,25(OH)₂D (15). VD metabolites are excreted in bile and reabsorbed in the terminal ileum. Therefore, conditions such as ileal disease, malabsorption syndromes, or intestinal resection can reduce serum 25(OH)D levels. The decline in VD metabolism among osteoporotic patients is frequently attributed to aging and diminished renal function. Aging reduces the skin’s capacity for VD synthesis, leading to lower circulating 25(OH)D, while renal impairment hampers conversion to the active 1,25(OH)₂D form (16). Hence, older adults and osteoporotic patients often require supplemental VD to counteract these physiological limitations. Interestingly, the American Endocrine Society recently recommended against routine screening or empirical VD supplementation in the general population (17), which further emphasizes the need to explore strategies that effectively elevate 1,25(OH)₂D levels to achieve its biological effects.

In this study, we identified *Bifidobacterium adolescentis* CCFM1447, a strain that significantly increases VD metabolite levels in fermentation supernatants, as demonstrated through an fecal fermentation model. We examined the effects of CCFM1447 on mice which have osteoporosis induced by retinoic acid (RA). Then we explored potential mechanisms underlying its action using Micro-CT scanning and analysis of intestinal microbiota. Findings of this study suggest that probiotics, such as *Bifidobacterium adolescentis* CCFM1447, have the potential to enhance VD activity levels, offering a promising approach for the management of osteoporosis.

## 2 Materials and methods

### 2.1 *In vitro* fermentation model screening strains

Dissolve feces in sterile PBS (1:10 w/v), mix thoroughly to homogenize. Filter the fecal slurry through two layers of gauze to remove insoluble particulate matter. This step is performed inside an anaerobic chamber. Prepare the fecal slurry. Using a 24-well plate, add 0.1 ml of bacterial solution and 0.3 ml of fecal slurry into each well. Then, add 1.6 ml of mGAM into each well. Incubate anaerobically for 36 h. Each sample is set up in triplicates. Use 0.4 ml of fecal slurry in some wells and 0.3 ml of fecal slurry and 0.1 ml of another bacterial strain in the blank controls. The entire 24-h fermentation process is conducted inside the anaerobic chamber. After fermentation, remove the 24-well plate and place it on ice for 15 min to stop fermentation. Transfer the fermented samples to 2 ml sterile centrifuge tubes. Centrifuge at 10,000 rpm for 10 min to separate the supernatant and pellet. Store the supernatant at −20°C. The levels of 25(OH)D and 1,25(OH)_2_D in the supernatant are detected by commercial assay kits.

### 2.2 Preparation of gavage strains for experimentation

The strain is stored in MRS medium supplemented with 30% (v/v) glycerol at −80°C. Before experimental use, the strain undergoes three consecutive activations. The bacterial suspension is centrifuged at 8,000 × *g* for 10 min to remove the supernatant. The pellet is washed three times with sterile physiological saline solution and resuspended in a solution of defatted milk powder at a concentration of 120 g/L. The resuspended culture is stored at −80°C. Before use, dilute the bacterial suspension with sterile physiological saline solution to a concentration of 10^9 CFU/ml for further applications. All strains used in this chapter were obtained from Culture collection of food microorganisms (CCFM), Biotechnology Centre of Jiangnan University. *Bifidobacterium adolescentis* CCFM1447 has been conserved in Guangdong Microbial Strain Conservation Centre (GDMCC No: 65382) on 31st October, 2024.

### 2.3 Mice experimental design

The study employed SPF-grade male C57BL/6 J mice, aged 7 weeks and weighing (20 ± 5) g, procured from Zhejiang Vital River Laboratory Animal Technology Co., Ltd. The animals were kept in a controlled environment at a temperature of (23 ± 2) °C and a relative humidity of (50 ± 10)%, with free access to standard rodent chow in accordance with national guidelines. All experiments were carried out at the Animal Experiment Center of Jiangnan University (Wuxi, China), with approval from the Jiangnan University Experimental Animal Ethics Committee (Ethics Approval No: JN.No20231030c1360128[515] and No: JN.No20241015c1041215[533]).

The volume of the gastric lavage solution was 200 μl. The bacterial solution was removed from the refrigerator and centrifuged to remove the supernatant. It was resuspended in saline to a final concentration of 1 × 10^9^ CFU/ml. This was used as the bacterial group gastric lavage solution.

#### 2.3.1 Experiment 1: CCFM1447 osteoporosis relief efficacy verification

Mice were acclimatized for 1 week. Afterward, they were randomly assigned to one of four groups (n = 6): Control, Model, VD, and CCFM1447. In 1–3 weeks, all groups, except the Control group, were orally gavaged with 90 mg/kg RA daily to induce osteoporosis. Following this induction period, mice were assigned to three groups: Model, VD, and CCFM1447. In 4–6 weeks, the VD group received a daily oral dose of 0.06 μg/kg of VD. The CCFM1447 group was treated with an oral gavage of *Bifidobacterium adolescentis* CCFM1447 suspension. The Control and Model groups were orally gavaged with saline daily.

#### 2.3.2 Experiment 2: individual effects of key differential strains

Mice were acclimatized for 1 week. They were then randomly assigned to one of eight groups (n = 6): Control, Model, Abx, Abx + Model, E.f, Abx + E.f, P.a, and Abx + P.a. In 1–3 weeks, all groups, except the Control, were given 90 mg/kg RA via oral gavage daily to induce osteoporosis. Concurrently, all antibiotic-treated groups were administered a combination of antibiotics during the last week of the modeling phase to mitigate intestinal microbiota disruption. The antibiotic cocktail included 100 mg/kg vancomycin, 200 mg/kg neomycin, 200 mg/kg ampicillin and 200 mg/kg metronidazole. In 4–6 weeks, the strain-treatment groups received an oral gavage of the corresponding bacterial strain. The remaining groups were given of saline.

At the conclusion of the study, fresh fecal samples were collected and stored at −80°C for microbiota diversity analysis. Mice were fasted for 12 h prior to being anesthetized with isoflurane, followed by euthanasia via orbital sinus puncture. Blood samples were quickly obtained from the orbital sinus, centrifuged at 5,000 *g* for 15 min at 4°C, and the serum was stored at −80°C. The femurs and tibias were carefully excised, connective tissues were removed, and the left bones were fixed in 4% paraformaldehyde in 5 ml sterile Eppendorf tubes for Micro-CT imaging. The right bones were stored at −80°C.

### 2.4 Determination of structural indices of mouse femur

After excising the right tibia from each mouse and fixing it in 4% paraformaldehyde, the sample was left to allow the liquid to evaporate for subsequent detection. The samples were then scanned using a Micro-CT scanner (Lateta LCT200, Hitachi-Aloka, Tokyo, Japan). The right femur from each mouse was properly positioned in the micro-CT imaging system for X-ray scanning. The scanning parameters were set as follows: 90 kV, 88 μA, a field of view of 18 mm, an acquisition time of 14 min, and a pixel size of 36 μm. Each femur underwent a 360° rotation to collect data, which were then imported into Analyze software (Version 12.0; AnalyzeDirect, Overland Park, KS, USA) for 3D reconstruction and analysis. A region of interest (ROI) 1 mm thick was selected starting 0.2 mm below the growth plate for bone parameter calculations. The following parameters were analyzed: Bone Mean (BM), Cortex Mean (CM), Trabeculae Mean (TM), trabecular separation (Tb.Sp), trabecular thickness (Tb.Th), trabecular number (Tb.N), trabecular connectivity density (Conn.D), bone surface (BS), bone volume (BV), and bone volume fraction (BV/TV).

### 2.5 Biochemical analysis

Serum calcium and alkaline phosphatase (ALP) levels were measured using commercial detection kits (Nanjing Jianjian Bioengineering Institute). Serum 25(OH)D and serum 1,25(OH)_2_D were measured using commercial Mouse ELISA kits (Beyotime Biotechnology, Shanghai, China). Osteocalcin (OCN) and Procollagen type I N-propeptide (PINP) levels were measured using commercial Mouse ELISA kits (E-EL-M0864, E-EL-M0233, Elabscience Biotechnology).

### 2.6 Metagenomic data processing and quality control

Metagenomic sequencing was conducted on the Illumina NovaSeq 6,000 platform (Illumina Inc., San Diego, CA, USA) at Beijing Nuohe Biomedical Technology Co., Ltd. (Beijing, China). The initial sequence data underwent preprocessing, which involved several steps: Trimmomatic (version 0.39) was used to eliminate low-quality reads (18). Sequences with an average base quality score lower than 30 were trimmed, and only sequences longer than 60 bp after trimming were retained as part of the high-quality output. The filtered sequences were then aligned to the human reference genome (GRCh38/hg38) using BWA (version 0.7.17), Samtools (version 1.9), and BEDTools (version 2.30.0), successfully removing any host-derived sequences from the data [H. (19, 20)].

### 2.7 Detection of metabolites in supernatant from *in vitro* single-bacterium culture

After the strain frozen at −80°C was taken out, it was connected to MRS liquid medium containing 0.05% (w/v) L-cysteine at 4% inoculum in an ultra-clean bench, and placed in an anaerobic chamber at 37°C for 18~ 24 h. Subsequently, the plate was scribed and a single colony was selected to the liquid medium, and the liquid was cultured for one generation, then strain preservation and identification were performed to ensure that there was no error in the identification and then activation was repeated, and the activation was repeated twice. After activation for two times, the culture was expanded, and the culture conditions were the same as above. After the activation and identification were completed, the culture was continued and incubated in an anaerobic chamber at 37°C for 16 h. VD3 was added to each tube of bacterial liquid to a final concentration of 10 μM. After incubation in the anaerobic chamber for 2 h, the bacterial liquid was taken out and centrifuged at 10,000 r/min for 10 min at 4°C, and the supernatant was retained at −80°C in the refrigerator for assaying, which would be reserved for the subsequent metabolite assay.

Remove the sample stored at low temperature, equilibrate to room temperature, accurately pipette 0.5 ml of the sample into a 2.0 ml Eppendorf centrifuge tube, add 1 ml of extraction solvent (hexane-ethyl acetate = 90:10), vortex vigorously for 1 min, and centrifuge at 4°C at 12,000 *g* for 15 min. Transfer 0.9 ml of the supernatant to a clean Eppendorf centrifuge tube, centrifuge at 37°C until dry, and redissolve the residue in 100 μl of methanol–water solution (methanol–water = 75:25).

Injection volume: 50 μl. Chromatography column: Phermon C18 column (Kinetex 2.6 μm, 100 × 3.0 mm). Mobile phase B: methanol solution containing 0.2 mM ammonium sulphate; mobile phase A: aqueous solution containing 0.2 mM ammonium sulphate. Flow rate: 0.5 ml/min. Detection time: 10 min. Column temperature: 40°C. The detection method used was ESI source positive ion mode MRM, with the MRM monitoring ion being m/z 423.1 → 369.0.

Prepare a series of mixed standard solutions by diluting the 1α,25(OH)₂D standard with methanol in a 1:1 ratio. Using Analyst 1.6.2 software, plot the standard solution concentration on the X-axis and the peak area of the standard as the Y-axis. A linear regression analysis was performed, and the regression equation was obtained using the ‘1/X^2^’ weighting. The peak area of the sample was substituted into the standard curve equation to calculate the concentration of 1α,25(OH)₂D in the serum sample.

### 2.8 Statistical analysis

Statistical and analytical evaluations were performed using GraphPad Prism 6 and SPSS software. Both control and treatment groups will be compared with the model group. Differences between groups will be assessed by one-way analysis of variance (ANOVA). A *p*-value of less than 0.05 was considered statistically significant. For macrogenomic data, analysis was carried out using the *psych* and *ggplot2* packages in R. * indicates significant differences from the model group: *p < 0.05, **p < 0.01, and ***p < 0.001.

**Figure 1:**
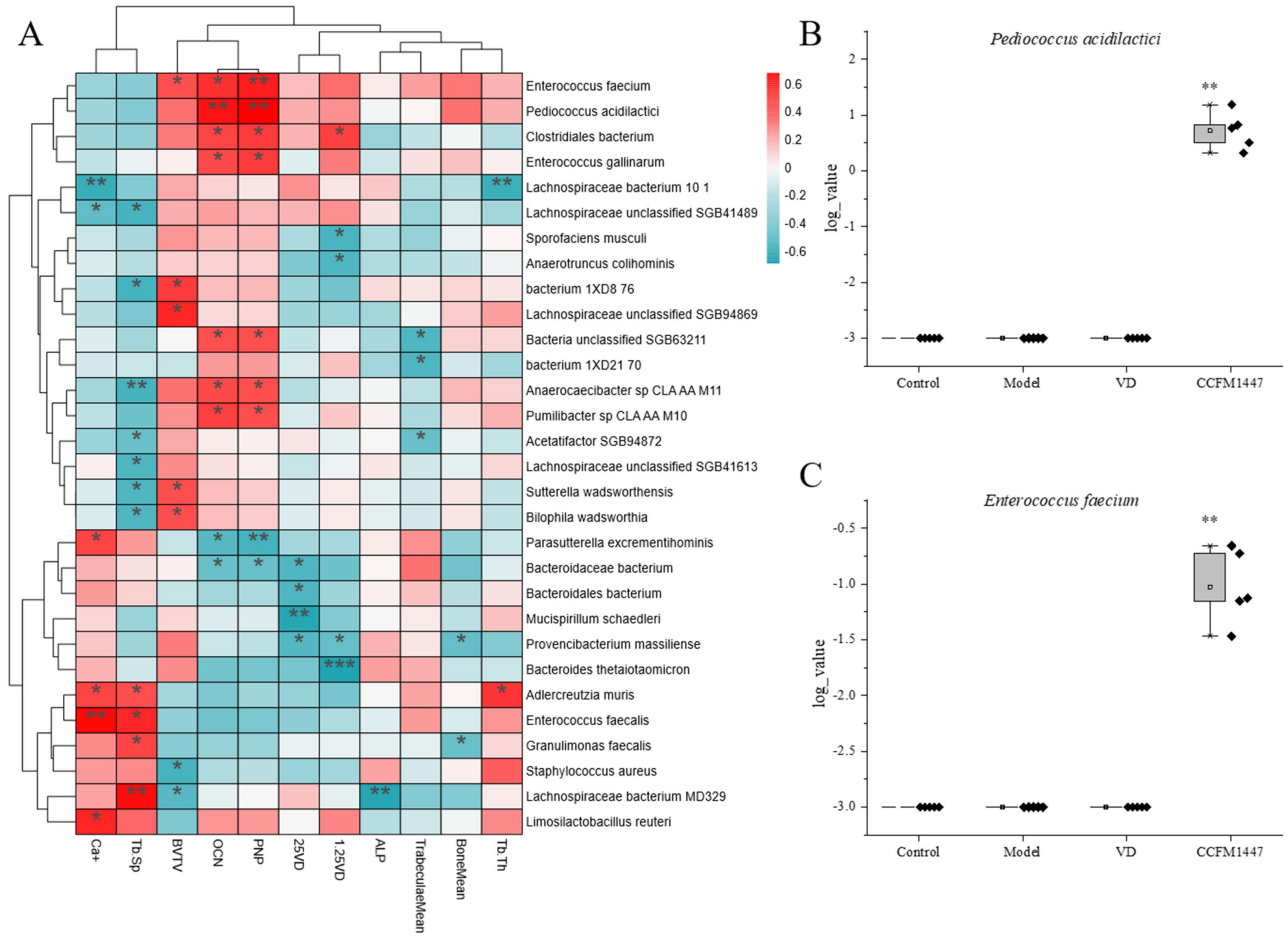
**(A)** Spearman’s correlation analysis of the relative abundance of gut microbial species with physiological and biochemical markers; Log-transformed representation of the relative abundance of **(B)** *Pediococcus acidilactici* and **(C)** *Enterococcus faecium* in different groups.* indicates significant differences from the model group: *p < 0.05, **p < 0.01, and ***p < 0.001.

Due to the skewed distribution of the raw microbial quantification data, data from Figures 1B,C were log-transformed (log10) to a base of 10 to meet the assumption of normality for subsequent statistical analyses. Undetected values were assigned a value of 0.001 for statistical analyses.

## 3 Results

### 3.1 Effect on VD metabolites in fermentation supernatants

We have found through recent research that some probiotics, especially *Bifidobacterium* and *Lactobacillus*, have the ability to improve bone health. We conjecture that this may be related to the promotion of VD metabolism by probiotics. Therefore, we selected some species of *Bifidobacterium* genus and *Lactobacillus* genus for *in vitro* fermentation experiments, including *Bifidobacterium longum*, *Bifidobacterium adolescentis*, and *Bifidobacterium. longum subsp. Infantis*, et al.

**Figure 2:**
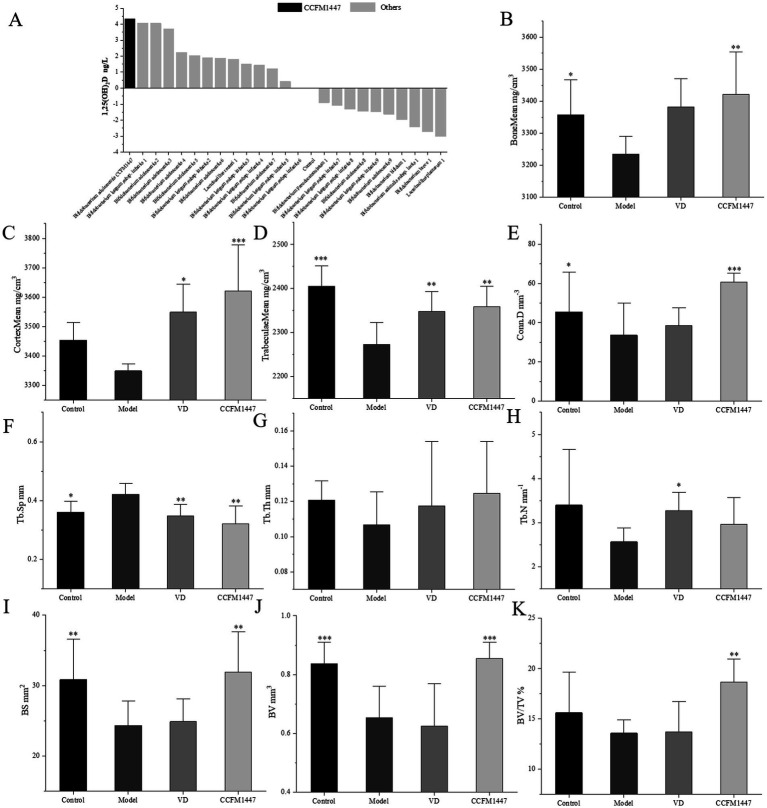
**(A)** Concentration of 1,25(OH)_2_D in fermentation supernatants. BMD analysis of **(B)** total, **(C)** cortical, and **(D)** trabecular bone in the distal femur. Micro-CT-based analysis of **(E)** Conn.D, **(F)** Tb.Sp, **(G)** Tb.Th, and **(H)** Tb.N. Additional bone parameters: **(I)** BS, **(J)** BV, and **(K)** BV/TV. * indicates significant differences from the model group: *p < 0.05, **p < 0.01, and ***p < 0.001.

A total of 23 strains from 10 different species bacterial were selected for in-vitro fecal fermentation, and the levels of the active vitamin D form, 1,25(OH)_2_D, in the supernatant were evaluated (Figure 2A). The addition of *Bifidobacterium adolescentis* CCFM1447 to the model resulted in a significant increase in the levels of the 1,25(OH)_2_D in the supernatant, when compared to the Control group, which did not receive bacterial inoculation.1,25(OH)₂D acts as a direct agonist of the VDR. Its binding regulates gene expression crucial for maintaining skeletal health. Additionally, it is utilized as a treatment for osteoporosis. Given its superior ability to elevate 1,25(OH)₂D levels, *B. adolescentis* CCFM1447 was chosen to explore its potential therapeutic effects on osteoporosis in mice.

### 3.2 Effects on bone structures

**Figure 3:**
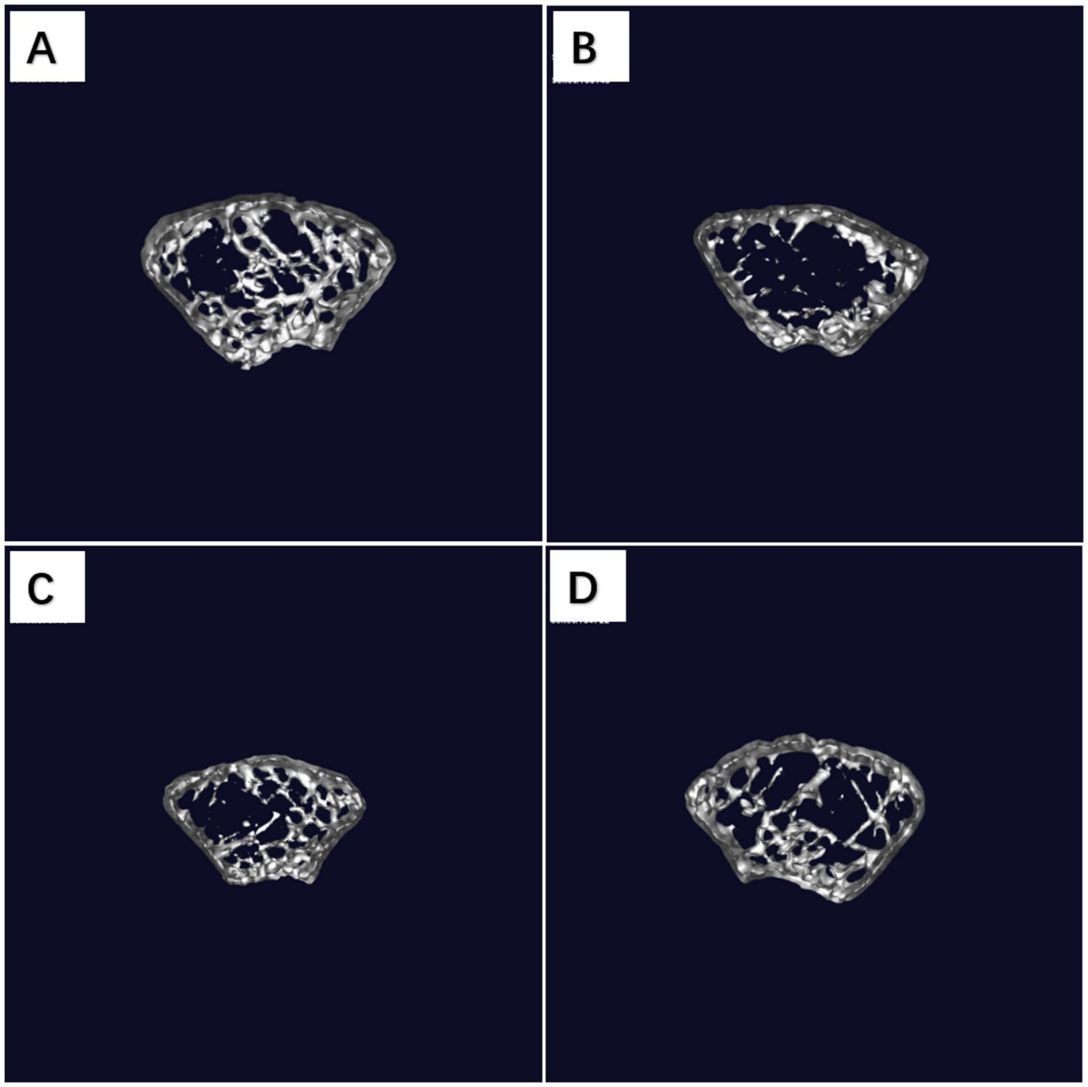
The three-dimensional reconstructions of mouse tibia. **(A)** Group Control; **(B)** group Model; **(C)** group VD; **(D)** group CCFM1447.

Bone mineral density (BMD) is the main indicator for the diagnosis of osteoporosis, and its value reflects the bone metabolism of the organism. After 3 weeks of modeling and 3 weeks of strain gavage intervention, the same parts of the distal femur of the mice were taken and examined by Mirco-CT scanning. The results showed that the BM and TM of mice in the Model group decreased significantly. It indicated the successful establishment of RA-induced osteoporosis model and poor natural recovery. After gavage intervention with CCFM1447 strain, the BM, CM and TM of mice were significantly increased. The results showed superior outcomes in the BM and CM indexes compared to the VD group. Further bone microstructural analysis is presented in Figures 2B–K. In the Model group, values for Tb.Th, Tb.N, and BV/TV were lower than those in the Control group. Conn.D, BS, and BV were significantly reduced, with a notable increase in TbSp. Following treatment with CCFM1447, Tb.Th and Tb.N showed an increase. Conn.D, BS, BV, and BV/TV were significantly enhanced, while Tb.Sp decreased significantly. VD supplementation led to significant improvements only in CM, TM, Tb.N and Tb.Sp. These results suggest that RA effectively induces osteoporotic changes and decreases BMD in mice, while CCFM1447 exhibited more pronounced beneficial effects compared to VD treatment in mitigating these conditions (Figure 3).

### 3.3 Effects on serum bone metabolism markers

Serum 25(OH)D levels serve as an indicator of the body’s VD reserves, while 1,25(OH)₂D is one of its most bioactive metabolites. The oral administration of 1,25(OH)₂D facilitates the absorption of calcium in the intestines, thereby addressing hypocalcemia and normalizing or reducing elevated serum ALP levels. Increased VD metabolites can also help lower plasma parathyroid hormone concentrations, contributing to improved bone mineralization. In the RA-induced osteoporotic mice, there was a significant reduction in serum levels of both 25(OH)D and 1,25(OH)₂D, indicating a decrease in both the storage and active forms of VD, impairing its physiological functions. However, after intervention with strain CCFM1447, the levels of 25(OH)D and 1,25(OH)₂D in the serum were significantly elevated compared to the Model group, suggesting that the strain enhanced VD metabolism.

**Figure 4:**
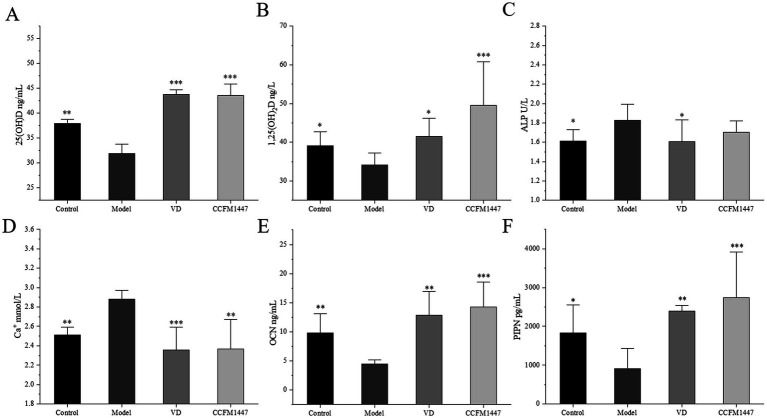
Effects on serum **(A)** 25(OH)D, **(B)** 1,25(OH)_2_D, **(C)** ALP, **(D)** Ca^+^, **(E)** OCN, **(F)** PINP concentration in mice. * indicates significant differences from the model group: *p < 0.05, **p < 0.01, and ***p < 0.001.

As shown in Figures 4C–F, RA-induced osteoporosis resulted in significantly higher ALP and calcium levels, indicating bone calcium loss and damage. This indicates that the retinoic acid-induced osteoporosis model in mice has been successfully established. Following intervention, the VD group exhibited a significant reduction in ALP and calcium levels. Similarly, CCFM1447 gavage notably reduced serum calcium, though ALP levels were only marginally decreased. Osteoporosis resulted in a noticeable decrease in OCN and PINP levels. The VD-treated group exhibited significantly higher levels of OCN and PINP than the Model group, indicating improved osteoblast function. Similarly, CCFM1447 gavage led to enhanced serum OCN and PINP levels, with a more pronounced improvement han VD treatment, highlighting its superior effectiveness in stimulating osteoblast activity.

### 3.4 Effects on the diversity of intestinal flora

**Figure 5:**
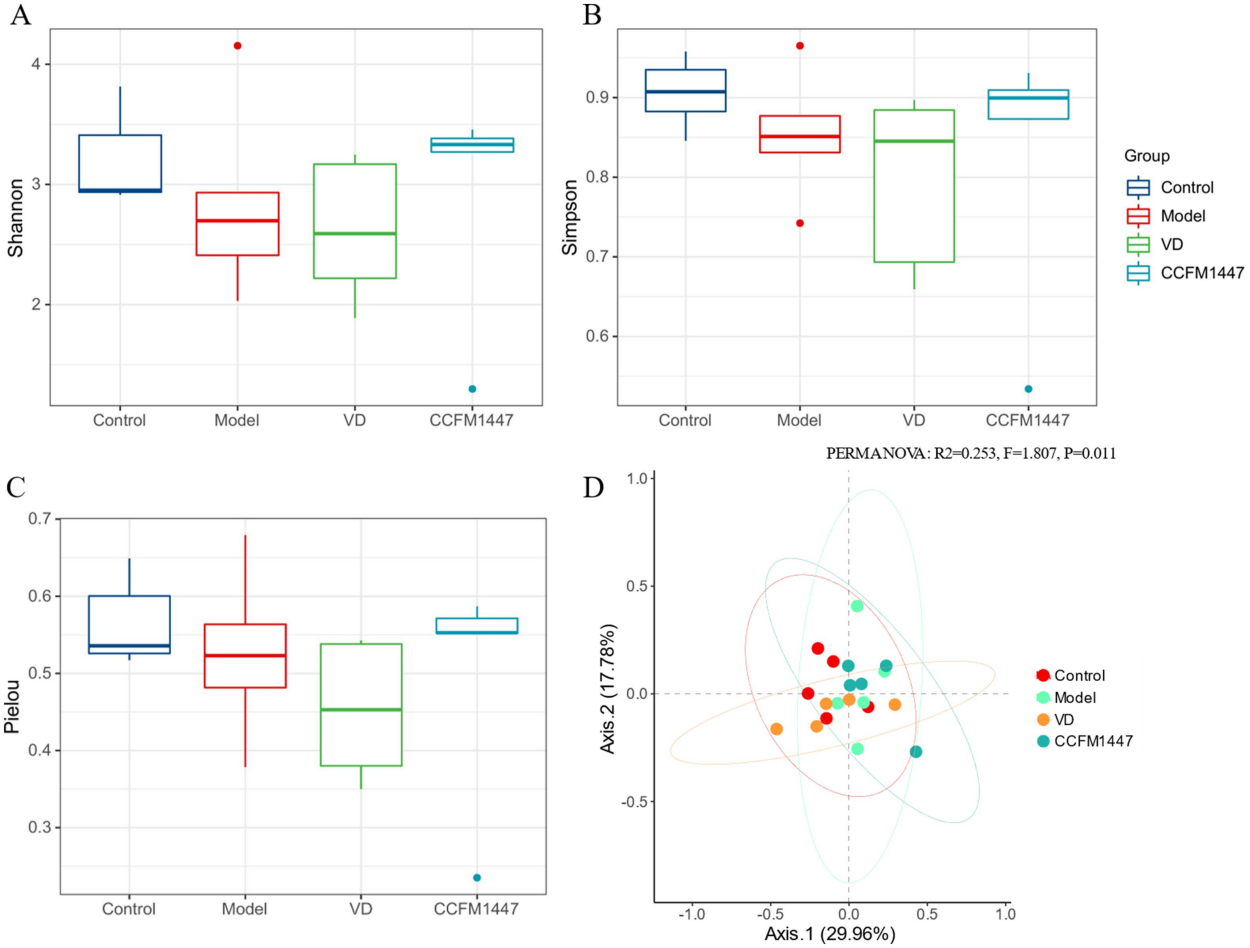
Species diversity analysis of intestinal flora across the different groups was conducted as follows: **(A)** Shannon index; **(B)** Simpson index; **(C)** Pielou index; **(D)** β-diversity based on PCoA algorithm.

Several previous studies have demonstrated that probiotics can influence the composition of the intestinal flora, with the potential to regulate its function. The alpha diversity indices (Shannon, Simpson, and Pielou) are presented in Figures 5A–C. However, the observed differences were not statistically significant. After VD and CCFM1447 gavage, there were no significant changes in Pielou’s homogeneity index, Shannon’s index, and Simpson’s index of the intestinal flora of mice. The reason why the diversity indices of mice in the VD and CCFM1447 groups were not significantly different from those of the model group may be related to the increase in the relative abundance of specific beneficial flora. This increase in selectivity modulates the structure of the intestinal flora as a whole and may lead to a relative decrease in other non-target microbiota, ultimately resulting in no significant change in the number of species types in the community.

This method helps to assess the differences or similarities in the intestinal flora composition between subjects. As shown in Figure 5D, the Principal coordinate analysis (PCoA) analysis revealed a marked difference in the intestinal flora structure between osteoporotic mice and healthy mice. Additionally, mice in the CCFM1447 and VD groups exhibited significant differences in microbiota composition compared to the Model group, suggesting that *Bifidobacterium adolescentis* CCFM1447 treatment effectively remodeled the intestinal flora structure in osteoporotic mice.

### 3.5 Effects on the structural composition of intestinal flora

**Figure 6:**
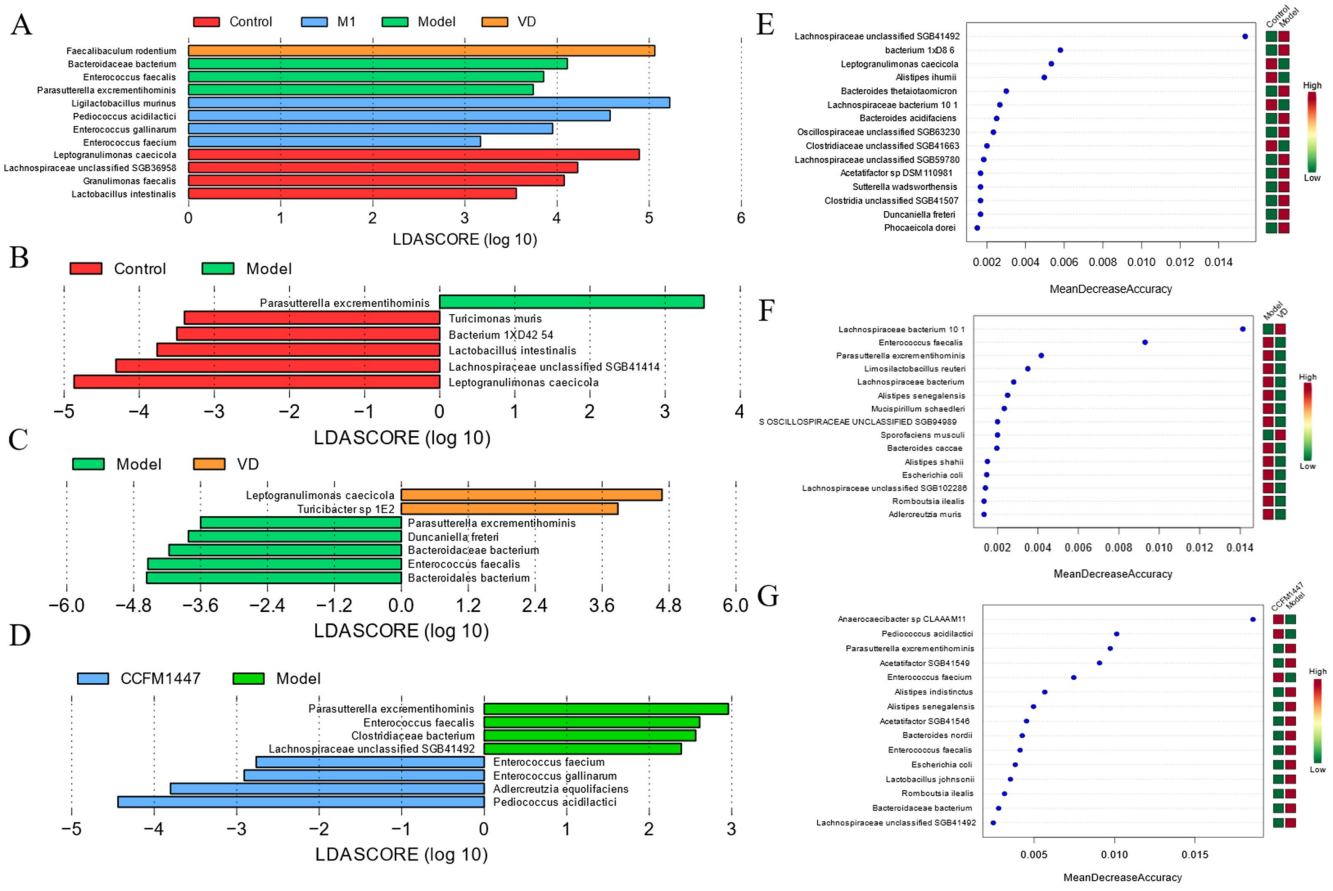
LEfSe analysis of fecal microbiota metagenomic species in mice: **(A,B,C,D)**; Importance analysis of key species characteristics based on the RF model: **(E,F,G)** (LDA > 3, *p* < 0.05).

The effects of VD and *Bifidobacterium adolescentis* CCFM1447 interventions on differential species of intestinal flora in osteoporotic mice were explored by LEfSe and RF analyses. As shown in Figure 6, same as the previous changes in relative abundance, there was a change in the relative abundance of *Faecalibaculum rodentium* in the VD group and *Pediococcus acidilactici* in the CCFM1447 group. And two-by-two comparison showed that there was a notable change in the relative abundance of *Parasutterella excrementihominis* in the intestinal flora of the model mice. These results suggest that VD and CCFM1447 decreased the relative abundance of some specific flora and increased the relative abundance of specific beneficial flora. This regulation of intestinal flora structure overall shifted the disrupted intestinal flora structure toward Control group mice. In contrast, VD and *Bifidobacterium adolescentis* CCFM1447 increased the relative abundance of specific beneficial flora in the intestinal flora and may also play a role in alleviating osteoporosis.

### 3.6 Correlation analysis of intestinal flora with physiological and biochemical markers

The correlation analysis of intestinal flora with physiological and biochemical markers is depicted in Figure 6. *Pediococcus acidilactici* was found to be positively correlated with beneficial indicators and negatively correlated with calcium loss and trabecular bone spacing. This aligns with the previously observed increase in *Pediococcus acidilactici* relative abundance following CCFM1447 intervention. An increase in the abundance of *Pediococcus acidilactici* may help alleviate osteoporosis symptoms. Conversely, the relative abundance of various Bacteroides species, including *Bacteroides thetaiotaomicron*, *Bacteroidales bacterium*, and *Bacteroidaceae bacterium*, showed a significant negative correlation with the levels of vitamin D metabolites, while being positively correlated with serum calcium levels and trabecular bone gap indices. These findings also corresponded to a notable decrease in the relative abundance of *Bacteroidales bacterium* after both vitamin D and CCFM1447 interventions. These results provide insight into the potential role of vitamin D and *Bifidobacterium adolescentis* CCFM1447 in modulating intestinal flora, offering a mechanism for their protective effects against osteoporosis through the regulation of specific microbial populations.

### 3.7 *Enterococcus faecalis* and *Pediococcus acidilactici* are probably the main strains in which CCFM1447 functions

Taking the intersection of LEfSe analyses, RF analyses, and association analyses of the differential groups, we found that CCFM1447 may promote VD transformation by enriching *Pediococcus acidilactici* and *Enterococcus feacium*. This was supported by the relative abundance in different groups as shown in Figures 1B,C. Gavage of CCFM1447 significantly increased *P. acidilactici* and *E. feacium* abundance compared to the model group. And this enrichment only appeared in the group with CCFM1447. This suggests that CCFM1447 may promote VD transformation and alleviate OP by specifically enriching *P. acidilactici* as well as *E. feacium*.

### 3.8 Functional validation of *Enterococcus faecalis* and *Pediococcus acidilactici*

**Figure 7:**
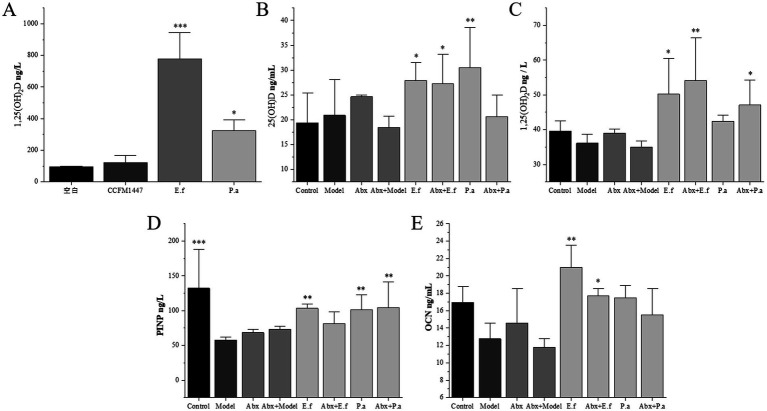
**(A)** Levels of 1,25(OH)_2_D in the supernatants of key differential bacteria after separate cultures; Effects of key differential flora on serum **(B)** 25(OH)D, **(C)** 1,25(OH)_2_D, **(D)** OCN, **(E)** PINP concentration in mice. * indicates significant differences from the model group: *p < 0.05, **p < 0.01, and ***p < 0.001.

Based on the key differential flora obtained in the above macrogenomic analysis, *in vitro* culture alone was performed, and the supernatant was taken for 1,25(OH)_2_D assay at the end. The results are shown in Figure 7A, there was no significant change in 1,25(OH)_2_D level in the supernatant of the medium of CCFM1447 after individual culture compared with the blank group. In contrast, both *Enterococcus faecalis* and *Pediococcus acidilactici* significantly increased the 1,25(OH)_2_D level in the supernatant of the culture medium. This suggests that CCFM1447 itself does not have the ability to transform VD, and may be able to increase the ability of the intestinal flora to transform VD by increasing the abundance of *E. faecalis* and *P. acidilactici* in the intestinal flora.

Therefore, we induced pseudo-sterile mice by quadruple antibiotic treatment to exclude the interference of intestinal flora for further functional evaluation and validation of key differential strains. The results are shown in Figures 7B–E. Compared with the Control group, the levels of 25(OH)D and 1,25(OH)_2_D were reduced in the Model group and Abx + Model group, but not significantly. This indicates that the intestinal flora has a certain VD conversion ability, and after the disappearance of intestinal flora, the main VD metabolism function still exists in the organism, although it will be affected to some extent. Meanwhile, the 1,25(OH)_2_D level was more stable, probably due to the body regulation brought about by osteoporosis symptoms, which induced the depletion of the storage form 25(OH)D to be converted into 1,25(OH)_2_D, thus maintaining the stability of 1,25(OH)_2_D level.

And compared to the model group, *E. faecalis*, Abx + *E. faecalis* and *P. acidilactici* group significantly increased the 25(OH)D and *E. faecalis*, Abx + *E. faecalis* and Abx + *P. acidilactici* group significantly increased the 1,25(OH)_2_D levels. This suggests that *E. faecalis* and *P. acidilactici* are able to function individually in transforming VD in the presence of removal of intestinal flora, with *E. faecalis* having the most significant effect, increasing both 25(OH)D and 1,25(OH)_2_D levels in Abx-treated mice. Similarly, the serum levels of type I procollagen amino-terminal procollagen peptide and osteocalcin were examined in the present study in all groups of mice to investigate the effect of key differential flora on the improvement of bone health in mice. Compared with the Control group, the PINP level was significantly reduced in the model group, and the OCN level was also reduced, but not significantly. And compared with the model group, the *E. faecalis*, *P. acidilactici*, Abx + *E. faecalis* and Abx + *P. acidilactici* group all showed more significant improvement in PINP and OCN levels. The above results further demonstrated that the mechanism by which CCFM1447 exerts its OP-relieving effect may be through the regulation of *E. faecalis* and *P. acidilactici* in the intestinal flora.

## 4 Discussion

RA has been shown to inhibit bone formation while promoting bone resorption. When administered to mice, RA induces a pathological process similar to osteoporosis, leading to altered bone structure and reduced bone density. In this model, significantly elevated serum ALP and calcium levels compared to the control group indicate extensive bone loss and confirm the successful induction of osteoporosis. Increased serum calcium levels serving as a marker for osteoporosis reflect indicating severe calcium depletion, reduced bone calcium reserves, and heightened bone turnover. Similarly, elevated ALP levels are associated with various bone disorders, including rickets, bone cancer, and bone metastases. OCN a hormone-like peptide secreted by osteoblasts, serves as a biochemical indicator of osteoblast activity. Approximately 20% of OCN produced by osteoblasts is released into the bloodstream, where its serum levels correlate with those in bone tissue, providing valuable insight into osteoblast function. Likewise, the expression of PINP, a marker of new bone formation, decreases as osteoblast activity diminishes, reflecting changes in the production of type I collagen.

*Bifidobacterium adolescentis* CCFM1447 was selected using an in-vitro fermentation model due to its ability to enhance vitamin D metabolite levels and alleviate osteoporosis symptoms. The effects of *Bifidobacterium adolescentis* CCFM1447 on bone structural parameters and serum biochemical markers were assessed in the RA-induced osteoporotic mice. The results demonstrated that *Bifidobacterium adolescentis* CCFM1447 significantly elevated the serum levels of 25(OH)D and 1,25(OH)₂D. These findings suggest that *Bifidobacterium adolescentis* CCFM1447 significantly mitigates the symptoms of RA-induced osteoporosis and plays a regulatory role in bone metabolism.

The alleviating effect of *Bifidobacterium adolescentis* CCFM1447 can be attributed to several key factors. First, VD helps with calcium uptake from the intestines into the bloodstream and directs calcium into the bones, especially benefiting calcium-deficient individuals such as children and older adults (21, 22). This function helps prevent excessive calcium loss from osteoblasts and reduces the risk of osteoporosis and bone development disorders in children. However, a large clinical trial revealed that VD supplementation did not significantly improve health outcomes like heart disease or cancer (23–26). Interestingly, some individuals are categorized as low, medium, or high responders to VD3 supplements, with 25% of people showing minimal response. This suggests that these low responders may require higher daily doses of VD than the standard recommendations (27–30). Furthermore, the body’s ability to metabolize VD is as important as VD supplementation (31).

*Bifidobacterium adolescentis* CCFM1447 has been shown to increase the levels of VD metabolites. Clinically, 25(OH)D levels are used to assess VD deficiency, with 25-hydroxy VD3 and VD2 being key indicators (31–33). The activation rate of VD is determined by the ratio of 1,25(OH)₂D to 25(OH)D, which helps gauge VD metabolism. Clinically, measuring 1,25(OH)₂D levels provides insights into the active VD status in conditions like metabolic bone disease (34). *Blautia* and *Ruminococcus* have the ability to metabolize VD, and the levels of active VD in the blood correlate with these microorganisms (35). This suggests that intestinal flora is importent in the bioavailability of VD, highlighting the potential of modulating the intestinal flora to enhance VD function and improve bone health.

VDR is present in various organs throughout the body. This makes 1,25(OH)₂D crucial to numerous physiological processes, as it can perform a variety of functions through the VDR, which is present throughout the body (36). 1,25(OH)₂D-VDR complex can activate the transcription of various genes involved in calcium absorption (37), parathyroid hormone (PTH), bone formation, and bone resorption. For example, genes regulating oxidative stress (38), calcium channels (e.g., TRPV6) (39), fibroblast growth factor 23 (FGF23), and calcium-binding proteins (e.g., calbindin-D28k) are activated (40), promoting calcium and phosphorus absorption, which benefits bone health. Additionally, the complex stimulates bone formation genes such as osteocalcin, promoting osteoblast activity and bone matrix synthesis, thereby enhancing bone density and strength (41). Moreover, the complex inhibits genes involved in bone resorption, such as RANKL and M-CSF, reducing osteoclast formation and minimizing bone destruction (42). The complex also regulates inflammation-related genes, such as NF-κB. The anti-inflammatory effects may help protect bone tissue and reduce osteoporosis symptoms (43).

In addition we detected that VD and *Bifidobacterium adolescentis* CCFM1447 intervened to significantly increase the relative abundance of *Adlercreutzia equolifaciens*, *Akkermansia muciniphila*, *pediococcus acidilactici* and Fae*calibaculum rodentium*. *Akkermansia muciniphila*, on the other hand, has been repeatedly reported to be associated with the gut barrier. Calcium absorption or VDR expression may be affected by the pro-inflammatory effects and damage on the intestine because of RA. *Akkermansia muciniphila* may play a role in this regard (44). *Pediococcus acidilactici* is a beneficial flora in both maintenance of intestinal flora diversity and intestinal homeostasis (44). *Faecalibaculum rodentium* plays a key role in modulating RA signaling to maintain eosinophil-dependent intestinal epithelial homeostasis, which contributes to the regulation of the intestinal barrier and overall homeostasis (45).

Additionally, 1,25(OH)₂D is recognized not only for its essential role in maintaining bone health, but also for its involvement in cell proliferation, differentiation, and immune function (46). Recent studies have emphasized the essential role of 1,25(OH)₂D as a key hormone in immune system regulation, similar to the Vitamin D Receptor (VDR), which is expressed across various organs. Additionally, the enzyme 1-alpha hydroxylase (CYP27B1), which is responsible for converting vitamin D into its active form, 1,25(OH)₂D, has been identified in several tissues beyond the kidneys. These include tumor cell supernatants, monocytes, macrophages, placenta, keratinocytes, and lymph nodes in individuals with nodal disease (36, 47). Notably, CYP27B1 expression has also been found in colon epithelial cells (48). Given the multifaceted roles of 1,25(OH)₂D in bone metabolism, immune modulation, and cellular differentiation, combined with the presence of CYP27B1 and VDR in the colon, it suggests a deeper and more intricate connection between gut health, intestinal barrier function, and the physiological effects of active vitamin D. This connection could provide insight into how *Bifidobacterium adolescentis* influences vitamin D metabolism in the gut by interacting with the intestinal flora. Moreover, the observed changes in intestinal flora after the intervention with *Bifidobacterium adolescentis* CCFM1447 appear to be closely linked to the regulation of gut barriers and homeostasis.

In this study, *Bifidobacterium adolescentis* CCFM1447 was experimentally found to have an excellent effect on improving OP symptoms and to enhance VD physiological activity by up-regulating the abundance of intestinal flora with the ability to transform VD. However, its mechanism of action to increase the abundance of these intestinal flora has not been clarified and will be further explored in depth in the future. In addition, the human body occupies a more dominant ability to metabolise VD, and further research is needed to improve VD physiological activity by regulating the human body and thereby increasing VD physiological activity. In this paper, we searched for key differential bacterial groups by analysing group-specific macrogenomes and non-target metabolomes and verified their functions by *in vitro* single-bacterial cultures, but the mechanism of transforming VD by differential bacterial groups is not clear. Therefore, in the future, these differential bacterial groups can be subjected to gene matching or transcriptome sequencing in order to further reveal their mechanism of transforming VD and lay a theoretical foundation for finding other strains with the ability to transform VD. These results provide a novel way of thinking about how probiotics affect VD metabolite levels by regulating intestinal flora to maintain bone health and gut homeostasis.

## 5 Conclusion

In conclusion, we identified a strain of *Bifidobacterium adolescentis* with the ability to enhance the activity of VD. The impact of this strain was further evaluated in a RA-induced osteoporotic mouse model. Significant bone loss and reduced serum VD metabolites were observed in the mice after 3 weeks of RA gavage. However, after 3 weeks of continuous *Bifidobacterium adolescentis* CCFM1447 intervention, we observed notable improvements in serum calcium, BMD, Tb. N, levels, decreased ALP levels, enhanced osteoblast activity, and increased VD metabolite levels, as well as modulation of intestinal flora. Compared to VD as a positive control, *Bifidobacterium adolescentis* CCFM1447 provided better improvements in osteoporosis-related symptoms. Additionally, the intervention’s effect on the intestinal flora revealed that CCFM1447, combined with VD, significantly increased the abundance of *Adlercreutzia equolifaciens*, *Akkermansia muciniphila*, *Pediococcus acidilactici*, and *Faecalibaculum rodentium*. These bacteria are closely linked to intestinal barrier function, the maintenance of gut microbiota balance, and possibly estramustine production. Given the multifaceted roles of 1,25(OH)₂D in bone health, immune modulation, and cellular differentiation, along with the presence of CYP27B1 and VDR in the colon, there is likely a more complex relationship between gut health, intestinal barrier integrity, and the physiological actions of vitamin D. This connection suggests that the metabolism of vitamin D, particularly its active form, may not only influence bone and immune functions but also play a critical role in preserving gut homeostasis and overall gastrointestinal health. Further studies are needed to test this hypothesis and evaluate whether these microbial species could be targeted to improve VD metabolism. Therefore, we verified their role in transforming vitamin D from both *in vitro* monoculture and antibiotic-treated model mice. We found that one possibility for CCFM1447 to enhance the physiological activity of VD is through up-regulation of the abundance of *Enterococcus faecalis* and *Pediococcus acidilactici* in the intestinal flora. These bacteria possess the capability to metabolize vitamin D and enhance the levels of its active metabolites, thereby mitigating symptoms of osteoporosis in mice. This offers a theoretical foundation for boosting vitamin D activity as a strategy to combat osteoporosis. The potential applications of this approach are significant, particularly in the development of products aimed at enhancing the physiological activity of vitamin D to prevent or treat osteoporosis and bone loss. This area holds great promise and warrants further investigation.

## Data Availability

The original contributions presented in the study are publicly available. This data can be found in here: https://www.ncbi.nlm.nih.gov/, accession number PRJNA1248171.

## References

[1] LeBoff MS Greenspan SL Insogna KL Lewiecki EM Saag KG Singer AJ. The clinician's guide to prevention and treatment of osteoporosis. Osteoporos Int. (2022) 33:2049–102. doi: 10.1007/s00198-021-05900-y, PMID: 35478046 PMC9546973

[2] Khan AA Morrison A Hanley DA Felsenberg D LK MC O'Ryan F. Diagnosis and management of osteonecrosis of the jaw: a systematic review and international consensus. J Bone Mineral Res. (2015) 30:3–23. doi: 10.1002/jbmr.2405, PMID: 25414052

[3] Crandall CJ Hovey KM Andrews C Cauley JA Stefanick M Shufelt C. Comparison of clinical outcomes among users of oral and transdermal estrogen therapy in the Women's Health Initiative observational study. Menopause (New York, NY). (2017) 24:1145–53. doi: 10.1097/GME.0000000000000899, PMID: 28697036 PMC5607093

[4] Shoback D Rosen CJ Black DM Cheung AM Murad MH Eastell R. Pharmacological management of osteoporosis in postmenopausal women: an Endocrine Society guideline update. J Clin Endocrinol Metab. (2020) 105:587–94. doi: 10.1210/clinem/dgaa048, PMID: 32068863

[5] Overman RA Borse M Gourlay ML. Salmon calcitonin use and associated Cancer risk. Ann Pharmacother. (2013) 47:1675–84. doi: 10.1177/1060028013509233, PMID: 24259626

[6] Kim SC Kim M-S Sanfélix-Gimeno G Song HJ Liu J Hurtado I. Use of osteoporosis medications after hospitalization for hip fracture: a cross-national study. Am J Med. (2015) 128:519–526.e511. doi: 10.1016/j.amjmed.2015.01.014, PMID: 25660252 PMC4414898

[7] Liu SK Munson JC Bell JE Zaha RL Mecchella JN Tosteson AN. Quality of osteoporosis care of older Medicare recipients with fragility fractures: 2006 to 2010. J Am Geriatr Soc. (2013) 61:1855–62. doi: 10.1111/jgs.12507, PMID: 24219186 PMC4084674

[8] Roerholt C Eiken P Abrahamsen B. Initiation of anti-osteoporotic therapy in patients with recent fractures: a nationwide analysis of prescription rates and persistence. Osteoporos Int. (2009) 20:299–307. doi: 10.1007/s00198-008-0651-x, PMID: 18551241

[9] Harrison SR Li D Jeffery LE Raza K Hewison M. Vitamin D, autoimmune disease and rheumatoid arthritis. Calcif Tissue Int. (2020) 106:58–75. doi: 10.1007/s00223-019-00577-2, PMID: 31286174 PMC6960236

[10] Latic N Erben RG. Vitamin D and cardiovascular disease, with emphasis on hypertension, atherosclerosis, and heart failure. Int J Mol Sci. (2020) 21:6483–3. doi: 10.3390/ijms21186483, PMID: 32899880 PMC7555466

[11] Roger B Despoina M Cliff R Katerina T Fernando R Brent RJ. The health effects of vitamin D supplementation: evidence from human studies. Nat Rev Endocrinol. (2021) 18:96–110. doi: 10.1038/s41574-021-00593-z, PMID: 34815552 PMC8609267

[12] Andrea H Carsten C. Time-resolved gene expression analysis monitors the regulation of inflammatory mediators and attenuation of adaptive immune response by vitamin D. Int J Mol Sci. (2022) 23:911–1. doi: 10.3390/ijms23020911, PMID: 35055093 PMC8776203

[13] Lea T Michael S Claudia P Michael F Viktor M Markus S. Cutaneous photosynthesis of vitamin D: an evolutionary highly-conserved endocrine system that protects against environmental hazards including UV-radiation and microbial infections. Anticancer Res. (2006) 26:2743–8. PMID: 16886686

[14] Cheng JB Levine MA Bell NH Mangelsdorf DJ Russell DW. Genetic evidence that the human CYP2R1 enzyme is a key vitamin D 25-hydroxylase. Proc Natl Acad Sci USA. (2004) 101:7711–5. doi: 10.1073/pnas.0402490101, PMID: 15128933 PMC419671

[15] Glorieux FH St-Arnaud R. Molecular cloning of (25-OH D)-1 alpha-hydroxylase: an approach to the understanding of vitamin D pseudo-deficiency. Recent Progress in hormone research (1998) 53:341–9. 9769714

[16] Latic N Erben RG. FGF23 and vitamin D metabolism. JBMR Plus. (2021) 5:e10558–8. doi: 10.1002/jbm4.10558, PMID: 34950827 PMC8674776

[17] Demay MB Pittas AG Bikle DD Diab DL Kiely ME Castro ML. Vitamin D for the prevention of disease: an Endocrine Society clinical practice guideline. J Clin Endocrinol Metab. (2024) 109:1907–47. doi: 10.1210/clinem/dgae290, PMID: 38828931

[18] Bolger AM Lohse M Usadel B. Trimmomatic: a flexible trimmer for Illumina sequence data. Bioinf (Oxf). (2014) 30:2114–20. doi: 10.1093/bioinformatics/btu170, PMID: 24695404 PMC4103590

[19] Li H. (2013). Aligning sequence reads, clone sequences and assembly contigs with BWA-MEM. Arxiv

[20] Quinlan AR Hall IM. BEDTools: a flexible suite of utilities for comparing genomic features. Bioinformatics. (2010) 26:841–2. doi: 10.1093/bioinformatics/btq033, PMID: 20110278 PMC2832824

[21] Weaver CM Gordon CM Janz KF Kalkwarf HJ Lappe JM Lewis R. The national osteoporosis foundation's position statement on peak bone mass development and lifestyle factors: a systematic review and implementation recommendations. Osteoporos Int. (2016) 27:1281–386. doi: 10.1007/s00198-015-3440-3, PMID: 26856587 PMC4791473

[22] Zhao JG Zeng XT Wang J Liu L. Association between calcium or vitamin D supplementation and fracture incidence in community-dwelling older adults: a systematic review and meta-analysis. JAMA. (2017) 318:2466–82. doi: 10.1001/jama.2017.19344, PMID: 29279934 PMC5820727

[23] Donlon CM LeBoff MS Chou SH Cook NR Copeland T Buring JE. Baseline characteristics of participants in the VITamin D and OmegA-3 TriaL (VITAL): effects on bone structure and architecture. Contemp Clin Trials. (2018) 67:56–67. doi: 10.1016/j.cct.2018.02.003, PMID: 29408561 PMC5877816

[24] LeBoff MS Chou SH Murata EM Donlon CM Cook NR Mora S. Effects of supplemental vitamin D on bone health outcomes in women and men in the VITamin D and OmegA-3 TriaL (VITAL). J Bone Miner Res. (2020) 35:883–93. doi: 10.1002/jbmr.3958, PMID: 31923341 PMC7217747

[25] LeBoff MS Murata EM Cook NR Cawthon P Chou SH Kotler G. VITamin D and OmegA-3 TriaL (VITAL): effects of vitamin D supplements on risk of falls in the US population. J Clin Endocrinol Metab. (2020) 105:2929–38. doi: 10.1210/clinem/dgaa311, PMID: 32492153 PMC7365686

[26] Manson JE Cook NR Lee IM Christen W Bassuk SS Mora S. Vitamin D supplements and prevention of Cancer and cardiovascular disease. N Engl J Med. (2018) 380:33–44. doi: 10.1056/NEJMoa1809944, PMID: 30415629 PMC6425757

[27] Carlberg C Seuter S de Mello VD Schwab U Voutilainen S Pulkki K. Primary vitamin D target genes allow a categorization of possible benefits of vitamin D₃ supplementation. PLoS One. (2013) 8:e71042. doi: 10.1371/journal.pone.0071042, PMID: 23923049 PMC3726591

[28] Jussi R Antonio N Tomi-Pekka T K VJ Sari V Tarja N. Changes in vitamin D target gene expression in adipose tissue monitor the vitamin D response of human individuals. Mol Nutr Food Res. (2014) 58:2036–45. doi: 10.1002/mnfr.201400291, PMID: 24975273

[29] Saksa N Neme A Ryynänen J Uusitupa M Mello VDF d Voutilainen S. Dissecting high from low responders in a vitamin D 3 intervention study. J Steroid Biochem Mol Biol. (2015) 148:275–82. doi: 10.1016/j.jsbmb.2014.11.012, PMID: 25448738

[30] Vukić M Neme A Seuter S Saksa N de Mello VD Nurmi T. Relevance of vitamin D receptor target genes for monitoring the vitamin D responsiveness of primary human cells. PLoS One. (2015) 10:e0124339. doi: 10.1371/journal.pone.0124339, PMID: 25875760 PMC4395145

[31] Orkaby AR Djousse L Manson JE. Vitamin D supplements and prevention of cardiovascular disease. Curr Opin Cardiol. (2019) 34:700–5. doi: 10.1097/hco.0000000000000675, PMID: 31425172 PMC7112175

[32] Holick MF Binkley NC Bischoff-Ferrari HA Gordon CM Hanley DA Heaney RP. Evaluation, treatment, and prevention of vitamin D deficiency: an Endocrine Society clinical practice guideline. J Clin Endocrinol Metab. (2011) 96:1911–30. doi: 10.1210/jc.2011-0385, PMID: 21646368

[33] Paweł P Beata K Mieczysław W Andrzej F Dorota Z Piotr S. Guidelines for preventing and treating vitamin D deficiency: a 2023 update in Poland. Nutrients. (2023) 15:695–5. doi: 10.3390/nu15030695, PMID: 36771403 PMC9920487

[34] Li Q Chan H Liu W-X Liu C-A Zhou Y Huang D. *Carnobacterium maltaromaticum* boosts intestinal vitamin D production to suppress colorectal cancer in female mice. Cancer Cell. (2023) 41:1450–1465.e8. doi: 10.1016/j.ccell.2023.06.011, PMID: 37478851

[35] Thomas RL Jiang L Adams JS Xu ZZ Shen J Janssen S. Vitamin D metabolites and the gut microbiome in older men. Nat Commun. (2020) 11:5997–7. doi: 10.1038/s41467-020-19793-8, PMID: 33244003 PMC7693238

[36] Adams JS Hewison M. Update in vitamin D. J Clin Endocrinol Metab. (2010) 95:471–8. doi: 10.1210/jc.2009-1773, PMID: 20133466 PMC2840860

[37] Haussler MR Jurutka PW Mizwicki M Norman AW. Vitamin D receptor (VDR)-mediated actions of 1alpha,25(OH)2vitamin D3: genomic and non-genomic mechanisms. Best Pract Res Clin Endocrinol Metabol. (2011) 25:543–59. doi: 10.1016/j.beem.2011.05.010, PMID: 21872797

[38] Emilio S Yaquelin HE José P. The role of vitamin D on redox regulation and cellular senescence. Free Radic Biol Med. (2022) 193:253–73. doi: 10.1016/j.freeradbiomed.2022.10.003, PMID: 36270517

[39] Meyer MB Watanuki M Kim S Shevde NK Pike JW. The human transient receptor potential vanilloid type 6 distal promoter contains multiple vitamin D receptor binding sites that mediate activation by 1,25-dihydroxyvitamin D3 in intestinal cells. Molecul Endocrinol. (2006) 20:1447–61. doi: 10.1210/me.2006-0031, PMID: 16574738

[40] van de Peppel J van Leeuwen JP. Vitamin D and gene networks in human osteoblasts. Front Physiol. (2014) 5:137. doi: 10.3389/fphys.2014.00137, PMID: 24782782 PMC3988399

[41] Terpening CM Haussler CA Jurutka PW Galligan MA Komm BS Haussler MR. The vitamin D-responsive element in the rat bone gla protein gene is an imperfect direct repeat that cooperates with other cis-elements in 1,25-dihydroxyvitamin D3-mediated transcriptional activation. Endocrinology. (1991) 5:373–85. doi: 10.1210/mend-5-3-373, PMID: 1653893

[42] Holliday LS Patel SS Rody WJ. RANKL and RANK in extracellular vesicles: surprising new players in bone remodeling. Extracell Vesicles Circ Nucleic Acids. (2021) 2:18–28. doi: 10.20517/evcna.2020.02, PMID: 33982033 PMC8112638

[43] Zeitelhofer M Adzemovic MZ Gomez-Cabrero D Bergman P Hochmeister S N'diaye M. Functional genomics analysis of vitamin D effects on CD4+ T cells *in vivo* in experimental autoimmune encephalomyelitis. Proc Natl Acad Sci USA. (2017) 114:E1678–87. doi: 10.1073/pnas.1615783114, PMID: 28196884 PMC5338504

[44] Flórez AB Vázquez L Rodríguez J Redruello B Mayo B. Transcriptional regulation of the Equol biosynthesis gene cluster in *Adlercreutzia equolifaciens* DSM19450T. Nutrients. (2019) 11:993–3. doi: 10.3390/nu11050993, PMID: 31052328 PMC6566806

[45] Grace CY Sena B Jannely V Madelyn M Eunyoung C Monia M. Faecalibaculum rodentium remodels retinoic acid signaling to govern eosinophil-dependent intestinal epithelial homeostasis. Cell Host Microbe. (2022) 30:1295–1310.e8. doi: 10.1016/j.chom.2022.07.015, PMID: 35985335 PMC9481734

[46] Liu PT Stenger S Li H Wenzel L Tan BH Krutzik SR. Toll-like receptor triggering of a vitamin D-mediated human antimicrobial response. Science. (2006) 311:1770–3. doi: 10.1126/science.1123933, PMID: 16497887

[47] Madhusmita M Danièle P Anna P Ferrez C-SP Michael K. Vitamin D deficiency in children and its management: review of current knowledge and recommendations. Pediatrics. (2008) 122:398–417. doi: 10.1542/peds.2007-1894, PMID: 18676559

[48] Lu Y Chen H Chen Y Zhao L Hou S. Accumulated LPS induced by colitis altered the activities of vitamin D-metabolizing hydroxylases and decreased the generation of 25-hydroxyvitamin D. Chem Biol Interact. (2024) 395:110997–7. doi: 10.1016/j.cbi.2024.110997, PMID: 38588969

